# Clinical insights into diabetic gastroparesis: gastric scintigraphy-based diagnosis and treatment outcomes

**DOI:** 10.1186/s12876-025-03977-x

**Published:** 2025-09-15

**Authors:** Mohamed-Naguib Wifi, Mohamed El-Sherbiny, Rasha Sobh Mohamed, Ahmed Kandeel, Sobhi Eid Rizk

**Affiliations:** 1 Internal Medicine Department, GEHepKA Unit, Cairo University, Cairo, Egypt; 2 Internal Medicine Department, Faculty of Medicine, Cairo University, Cairo, Egypt; 3 Nuclear Medicine Department, Cairo University, Cairo, Egypt

**Keywords:** Diabetes mellitus, Gastric scintigraphy, Gastroparesis, G-POEM

## Abstract

**Background:**

Long-standing diabetes mellitus (DM) can lead to macrovascular and microvascular complications, including autonomic neuropathy, which disrupts gut motility. Gastroparesis (GP) is defined as delayed gastric emptying of solids (with or without liquids) in the absence of any mechanical obstruction. The gold standard test for diagnosing gastroparesis is gastric scintigraphy (GS) using a solid meal. Gastroparesis poses diagnostic and therapeutic challenges, and can significantly impact patients with DM. The purpose of this study is to evaluate the prevalence of gastroparesis among symptomatic patients and assess treatment outcomes, with particular focus on identifying clinical predictors of delayed gastric emptying and factors associated with response to medical therapy in confirmed cases.

**Patients and methods:**

From June 2022 to June 2024, all patients visiting the diabetes clinic in Cairo University Hospital for any reason were screened for symptoms of gastroparesis using the gastroparesis cardinal symptom index (GCSI). Symptomatic patients underwent solid gastric scintigraphy. Those diagnosed with GP were treated for three months and refractory cases were offered G-POEM.

**Results:**

Thirty-two patients with moderate-to-severe symptoms of gastroparesis were the population of this study. Of these, 62.5% had delayed gastric emptying on solid gastric scintigraphy. A GCSI > 23 independently predicted delayed gastric emptying on solid gastric scintigraphy (OR 1.153, 95% CI (1.009–1.317), *p* = 0.036). 55% of GP patients achieved improvement in symptoms after three months of optimized medical therapy, and two out of four cases had sustained improvement for one year after G-POEM. The responders to medical treatment were significantly older, had lower GCSI and greater reduction in hemoglobin A1c (HbA1c) compared to those in the refractory group (*p* = 0.046, 0.012, 0.012, respectively).

**Conclusion:**

This study highlighted the role of the GCSI in assessing and monitoring gastroparesis, particularly in resource-limited settings. Diabetic GP differs in clinical presentation and management from other types of GP. Optimizing glycemic control may contribute to symptoms improvement. Older age and lower symptoms burden at presentation may help identify patients more likely to benefit from medical therapy.

## Background

Long-standing DM leads to many macrovascular and microvascular complications, including autonomic neuropathy, which disrupts gut motility. Gut dysmotility in DM occurs via several mechanisms, including destruction of interstitial cells of Cajal (which is the pacemaker of the enteric neuronal plexuses), and reduction of neuronal nitric oxide synthase, leading to gastric dysmotility, pyloric spasm, and eventually delayed gastric emptying [1]. Gastroparesis, defined as delayed gastric emptying of solids (with or without liquids) after exclusion of mechanical obstruction, is a well-recognized but often underdiagnosed complication of DM. Its most frequent symptoms are nausea, vomiting, early satiety, post-prandial fullness, bloating, and abdominal pain [2].

The GCSI is a validated tool for assessing symptoms of gastroparesis using a 6-point Likert response scale (from 0 to 5). It evaluates the following symptoms in the past two weeks: nausea, retching, vomiting, stomach fullness, inability to finish a meal, excessive fullness, loss of appetite, bloating, and abdominal distension [3]. The gold standard for diagnosing gastroparesis is gastric scintigraphy which demonstrates delayed gastric emptying of a solid meal [4]. Treatment options for GP include dietary modifications, anti-emetics, and proton pump inhibitors (PPIs). Per-oral endoscopic myotomy (POEM), initially developed for achalasia, is a promising new technique for refractory gastroparesis. It offers shorter operative time, shorter post-procedure hospital stay, and fewer adverse events compared to laparoscopic pyloroplasty [5].

Despite recognition of gastroparesis in clinical guidelines, significant variability remains in its clinical presentation and in the correlation between symptoms and objective measures of delayed gastric emptying [6]. Furthermore, the clinical predictors of treatment response are poorly defined. This prospective observational study aims to evaluate the prevalence of gastroparesis among symptomatic patients and assess treatment outcomes, with particular focus on identifying clinical predictors of delayed gastric emptying and factors associated with response to medical therapy in confirmed cases.

## Patients and methods

### Inclusion and exclusion criteria

Patients aged ≥ 18 years with type 1 or type 2 DM for at least five years were screened for gastroparesis using GCSI at Cairo University Hospital’s diabetes clinic from June 2022 to June 2024. Patients were included if they had a GCSI of ≥ 10 and persistent symptoms for at least one month. Since gastroparesis and neuropathy typically develop over several years in DM, the five-year DM duration criterion was set to reduce the likelihood of including idiopathic or post-viral gastroparesis. Participants were excluded if they had any of the following: history of gastric surgery, organic gastrointestinal disease, malignancy, chronic debilitating diseases, using glucagon-like peptide-1 agonists or opioids, neuro-muscular diseases like parkinsonism, scleroderma, and pregnancy.

### Symptoms assessment

Gastroparesis symptoms were assessed using the GCSI, which consists of 9 items rated on a 0–5 Likert scale (0 = no, 1 = very mild, 2 = mild, 3 = moderate, 4 = severe, 5 = very severe). Since a validated Arabic version of the GCSI questionnaire was not available, the English version was verbally translated into Arabic to ensure patient comprehension. Patients completed the GCSI questionnaire by rating the severity of each of the 9 symptoms over the past two weeks: nausea/vomiting subscale (nausea, retching, and vomiting), post-prandial fullness/early satiety subscale (stomach fullness, inability to finish a normal-sized meal for a healthy person, excessive post-prandial fullness, and loss of appetite), and bloating/distention subscale (bloating and visible abdominal distension). Total GCSI was the sum of all 9 items. A GCSI score from 10 to 19 was considered moderate, and ≥ 20 was considered severe. Average GCSI was calculated by calculating the mean of the three subscales.

### Clinical evaluation

Eligible patients were subjected to a thorough history taking and clinical examination, including:

- Type and duration of DM.
- Anti-diabetic medications.
- DM complications:

1. Peripheral neuropathy: history of tingling, numbness, or hypoesthesia.
2. Diabetic nephropathy: either microalbuminuria (urinary albumin/creatinine ratio ≥ 30 mg/g or estimated glomerular filtration rate (eGFR) < 60 ml/min/1.73m^2^ using chronic kidney disease epidemiology collaboration (CKD-EPI) creatinine 2021 equation.
3. Retinopathy: history of diabetic retinopathy detected by fundus examination.
4. History of previous myocardial infarction, anginal symptoms, cerebrovascular stroke, peripheral vascular disease, and diabetic foot.

- Comorbidities: hypertension and thyroid disorders.
- Body mass index (BMI) was calculated as body weight in kilograms (kg) divided by the square of height in meters (m^2^). BMI between (18.5 and 24.9) kg/m² was considered normal, (25–29.9) kg/m² was considered overweight, and ≥ 30 kg/m² was considered obese.
- Diabetic dyslipidemia was defined as total cholesterol > 200 mg/dL, low-density lipoprotein (LDL) > 70 mg/dL, or triglycerides > 150 mg/dL.

### Laboratory tests

Blood samples were taken for:

- HbA1c (%): <7% was considered good control, (7–8.9%) fair control, (9–10.9%) poor control, and ≥ 11% very poor control.
- TSH, CBC, kidney and liver function tests, urinary albumin/creatinine ratio, and lipid profile.

### Gastric scintigraphy

Eligible patients were invited to undergo solid gastric scintigraphy, but six patients declined the procedure and were excluded. Baseline characteristics of those who declined scintigraphy were not analyzed but their total number was recorded to assess the impact of potential selection bias. Gastric emptying was assessed after ingestion of 300 g sandwich meal containing 1 mCi of 99 m Tc-DTPA-labeled boiled eggs. Any medication affecting gastric motility (prokinetics, and anticholinergics) was stopped for 48 h prior to testing. The time activity curve obtained from the geometric mean of gastric counts displayed for all time points was constructed. The gastric emptying half-time (T_1_/_2_) for a solid meal was computed by interpolation from the observed data. Gastroparesis was defined as delayed gastric emptying (T_1_/_2_ ≥90 min). The GP group included patients with GP symptoms and delayed gastric emptying, while the GP-like group included patients with GP symptoms and normal gastric emptying.

### Medical treatment for the gastroparesis group

All patients in the GP group were subjected to:

- Dietary modifications (frequent small semisolid meals and low-fat, low-fiber diet),
- Blood glucose optimization (intensified insulin regimens),
- PPIs and prokinetics (domperidone 10 mg, three times daily), and.
- Discontinuation of dipeptidyl peptidase-4 inhibitors (DPP-4i)/Metformin combination.

Monthly follow-up was conducted to ensure patients compliance, modify insulin regimens (if needed), and monitor the response to treatment. Patients were assessed after three months using HbA1c (%) and GCSI. Gastric scintigraphy was not repeated after treatment, as the primary focus of this study was symptoms improvement rather than changes in gastric scintigraphy parameters. Response to treatment was defined as a ≥ 40% reduction in the total GCSI from the baseline or ≥ 9-point reduction, along with patient-reported satisfaction with symptoms improvement. Patients were considered refractory if they had persistent symptoms despite optimized medical therapy for at least three months, including dietary modifications, prokinetic agents, and antiemetics after exclusion of gastric outlet obstruction using gastroduodenoscopy.

### G-POEM for refractory patients

Patients meeting refractory criteria were offered G-POEM, performed by an experienced endoscopist in POEM under general anesthesia and tracheal intubation. G-POEM consisted of four principal steps: (1) submucosal injection followed by mucosal incision 4–5 cm proximal to the pyloric canal, (2) creation of a submucosal tunnel towards a pyloric ring, (3) a complete myotomy 2–3 cm long, (4) closure of the incision with endoscopic clips. Patients were admitted post-procedure until recovery. They were followed up at 3-, 6- and 12-months post-procedure to assess complications and symptoms improvement.

Patients in the GP-like group were treated according to guidelines for functional dyspepsia, and they will be followed to assess the potential development of delayed gastric emptying over time.

### Statistical methods

Data were coded and entered using the statistical package for the Social Sciences (SPSS) version 28 (IBM Corp., Armonk, NY, USA). Data was summarized using mean, standard deviation, median, minimum, and maximum in quantitative data and using frequency (count) and relative frequency (percentage) for categorical data. Comparisons between quantitative variables were done using the non-parametric Mann-Whitney test. For comparing categorical data, Chi square (χ2) test was performed. Exact test was used instead when the expected frequency is less than 5. Correlations between quantitative variables were done using Spearman correlation coefficient. Multivariate stepwise logistic regression analysis was done to detect independent predictors of gastroparesis, and response to medical treatment. It was initially conducted by including all significant parameters from the univariate analysis. After stepwise selection (both forward and backward), only one variable remained in the final model.

## Results

Thirty-two patients with moderate-to-severe symptoms of gastroparesis were included in the study. Among them, 20 patients (62.5%) had delayed gastric emptying (GP group), while 12 patients (37.5%) had normal gastric emptying (GP-like group). Table 1 presents the demographic, clinical, and laboratory data of the study population. The majority of patients were females (81.3%), with a mean age of 40.59 ± 11.13 years. Type 2 DM was more common (78%, 25 patients), while type 1 DM represented 28% (7 patients). The mean duration of DM was 12.44 ± 5.44 years. There were no significant differences between GP and GP-like groups regarding the age, sex, type of DM, DM treatment or complications, HbA1c (%), smoking status, or other comorbidities. Although not statistically significant, diabetic nephropathy was more prevalent in the GP group than the GP-like group (12 vs. 3 cases, *p* = 0.055).

**Table 1:** The demographic, clinical, and laboratory data of all study population, the GP group, and the GP-like group

Variables	All population		GP group		GP-like group		*P* value
	Mean	SD	Mean	SD	Mean	SD	
Age (Y)	40.59	11.13	40.70	11.14	40.42	11.60	0.893
Duration of DM (Y)	12.44	5.44	13.00	5.79	11.50	4.91	0.477
Duration of insulin (Y)	11.28	6.12	12.22	7.12	9.88	4.29	0.305
BMI	30.87	5.95	30.41	6.82	31.63	4.29	0.307
HbA1c (%)	9.35	2.29	9.24	2.04	9.54	2.75	0.985
TSH	2.73	2.32	2.78	2.19	2.64	2.62	0.795
Hb	12.12	1.80	11.77	1.91	12.72	1.49	0.216
TLC	6.47	2.44	6.73	2.88	6.03	1.44	0.899
PLT	280.67	64.23	285.11	72.41	273.00	49.26	0.735
S. Creatinine	0.89	0.41	0.99	0.47	0.73	0.18	0.083
eGFR	100.97	26.69	94.95	29.43	111.00	18.34	0.170
AST	28.76	14.15	31.75	14.79	19.20	5.54	**0.032**
ALT	22.50	9.46	24.07	10.25	19.14	6.96	0.368
uACR	98.06	83.40	123.60	93.17	55.50	42.04	0.093
TC	208.47	42.42	208.17	45.33	208.92	39.60	0.917
LDL	112.50	44.39	106.79	45.81	122.50	42.82	0.402
HDL	55.86	27.13	48.27	15.67	68.50	38.19	0.181
TG	167.90	117.15	158.28	108.30	182.33	132.94	0.573

SD Standard Deviation, Y Years, BMI Body Mass Index, TLC Total Leukocyte Count, Hb Hemoglobin, PLT Platelet Count, S. Creatinine Serum Creatinine, eGFR Estimated Glomerular Filtration Rate, AST Aspartate Aminotransferase, ALT Alanine Transaminase, uACR Urinary Albumin/Creatinine Ratio, TC Total Cholesterol, LDL Low-Density Lipoprotein, HDL High-Density Lipoprotein, TG Triglycerides

### Symptoms of gastroparesis

**Fig. 1:**
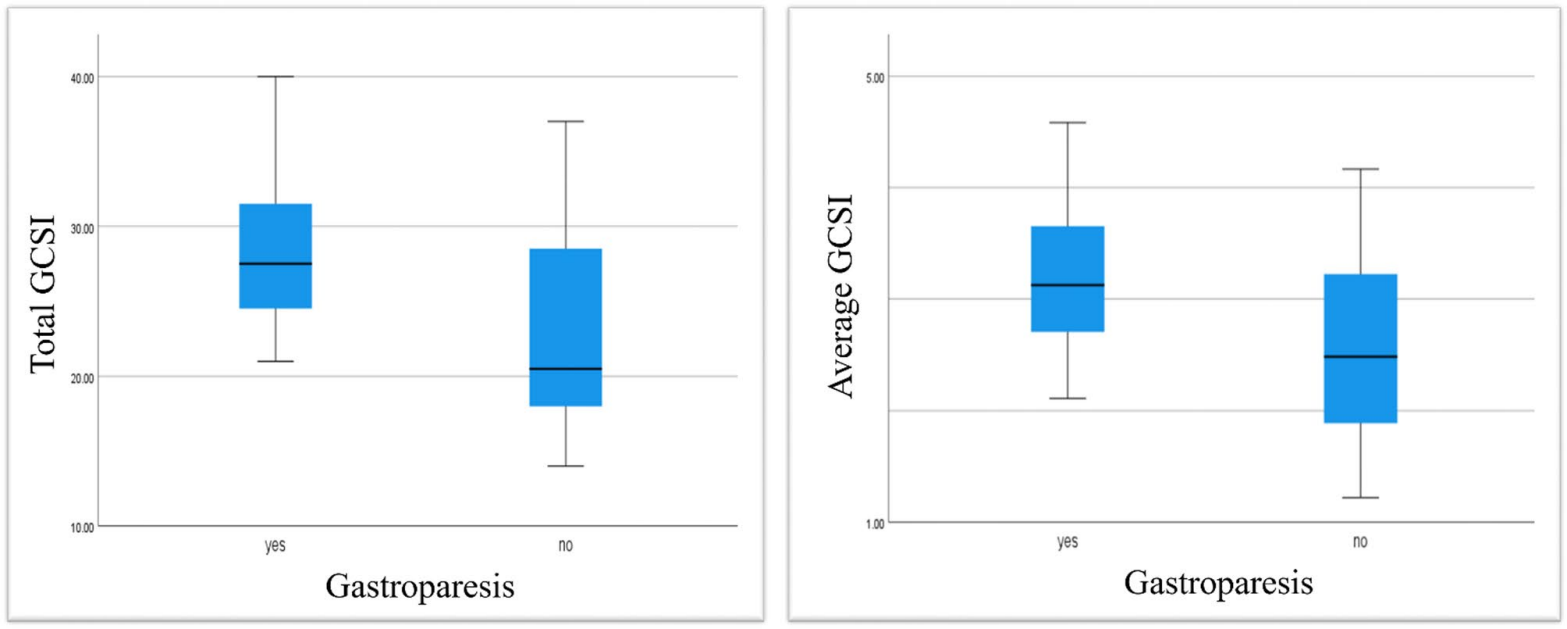
The severity of symptoms according to the total and the average GCSI in the GP group versus the GP-like group

This study found that 12.5% of cases had moderate symptoms, while 87.5% had severe symptoms. The mean duration of symptoms was 41.03 ± 72 months. The mean total GCSI in all study populations, GP group, and GP-like group were 26.41 ± 6.91, 28.50 ± 5.77, and 22.92 ± 7.46, respectively. As shown in Table 2, the dominating symptom in all groups was post-prandial fullness, while bloating/distension subscale was the most dominant subscale. The GP group had significantly more severe symptoms than the GP-like group according to the total GCSI (*p* = 0.021) and the average GCSI (*p* = 0.048) (Fig. 1).

**Table 2:** Symptoms of GP in all study populations, the GP, and the GP-like groups

Symptoms	All populations		GP group		GP-like group		*P* value
	Mean	SD	Mean	SD	Mean	SD	
Symptoms Duration (M)	41.03	72.00	53.30	88.43	20.58	19.28	0.366
Total GCSI /45	26.41	6.91	28.50	5.77	22.92	7.46	**0.021**
Average GCSI /5	2.96	0.85	3.20	0.69	2.55	0.96	**0.048**
N/V subscale/5	2.25	1.30	2.47	1.35	1.89	1.17	0.255
PPF/ ES subscale/5	3.21	0.91	3.41	0.89	2.88	0.88	0.091
B/D Subscale/5	3.41	1.65	3.73	1.42	2.87	1.92	0.182
Nausea	3.06	1.52	3.05	1.70	3.08	1.24	0.774
Retching	2.00	1.70	2.25	1.71	1.58	1.68	0.307
Vomiting	1.69	1.89	2.10	2.02	1.00	1.48	0.146
Stomach fullness	3.84	1.42	3.95	1.36	3.67	1.56	0.604
Not able to finish a meal	3.22	1.54	3.55	1.28	2.67	1.83	0.170
Excessive post-prandial fullness	3.09	1.65	3.30	1.59	2.75	1.76	0.387
Loss of appetite	2.69	1.75	2.85	1.81	2.42	1.68	0.477
Bloating	3.44	1.68	3.80	1.44	2.83	1.95	0.158
Visible abdominal distension	3.37	1.70	3.65	1.50	2.92	1.98	0.346

SD Standard Deviation, M Months, GCSI Gastroparesis Cardinal Symptom Index, N/V Nausea/ Vomiting, PPF/ES Post-prandial Fullness/Early Satiety, B/D Bloating/Distension

**Fig. 2:**
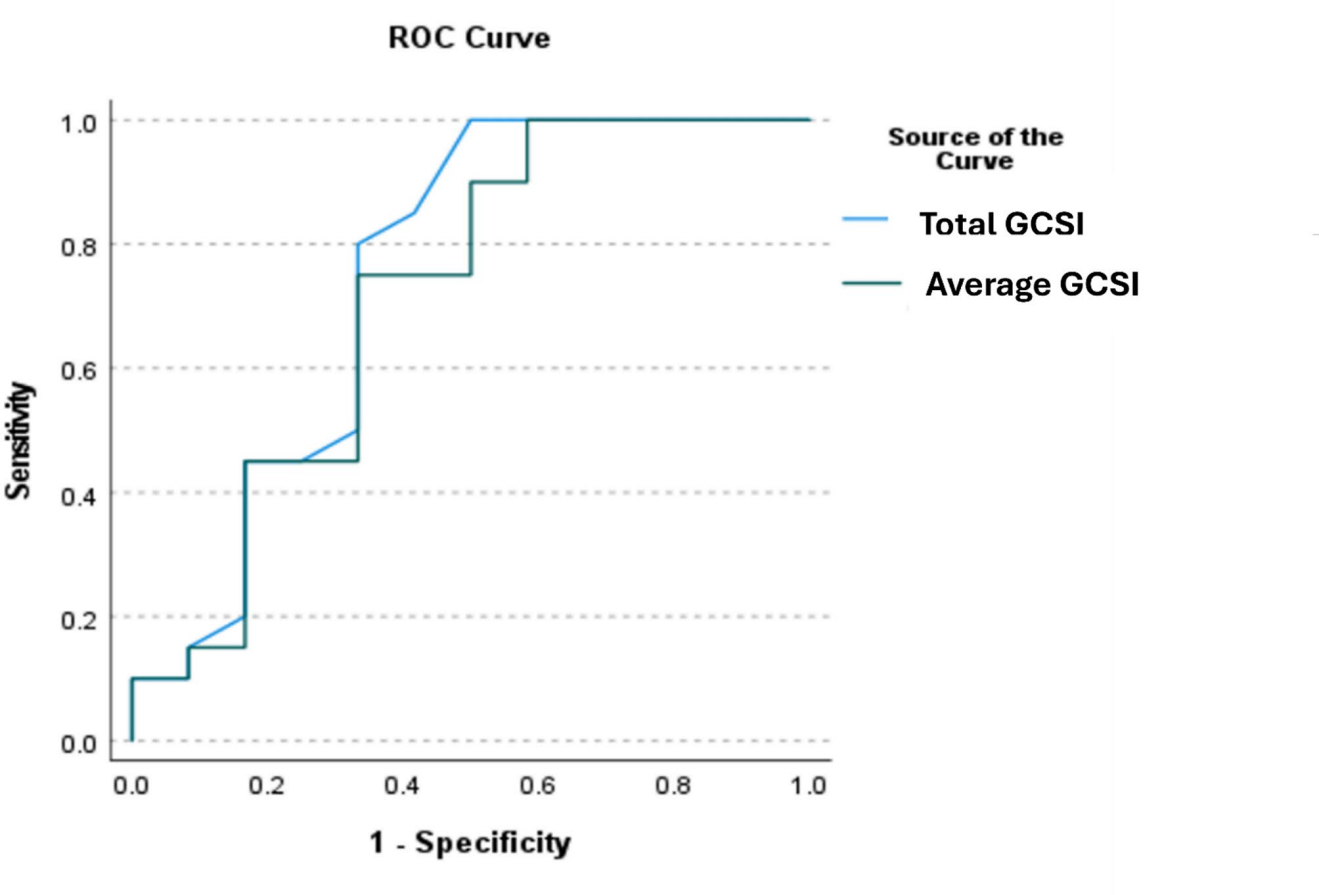
The ROC curve for the total and the average GCSI in predicting the presence of delayed gastric emptying

The logistic regression analysis revealed that the total GCSI is an independent predictor of delayed gastric emptying on solid gastric scintigraphy (OR 1.153, 95% CI (1.009–1.317), *p* = 0.036). A total GCSI greater than 23 demonstrated 80% sensitivity and 66.7% specificity for the delayed gastric emptying (AUC 0.746, *p* = 0.016, 95% CI 0.545–0.947) (Fig. 2).

### Scintigraphy parameters

**Fig. 3:**
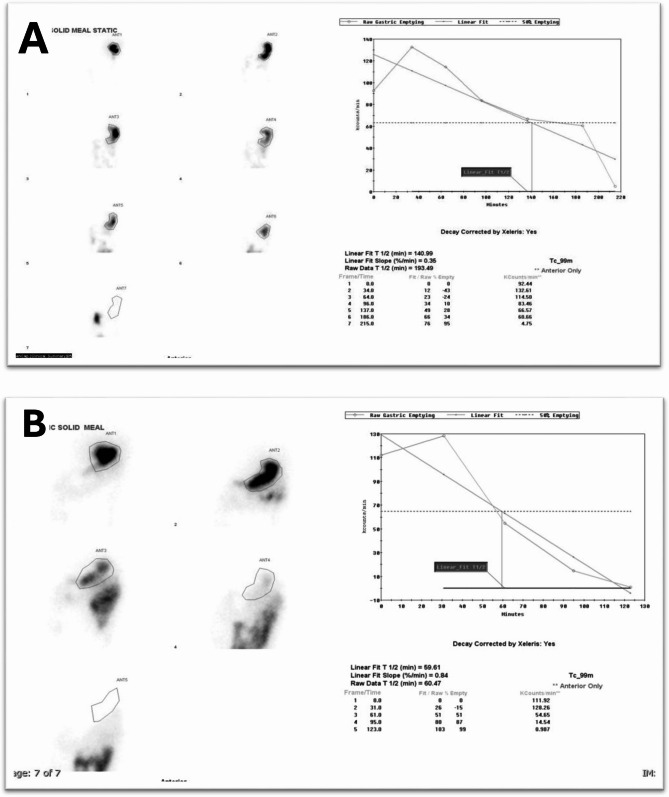
Delayed gastric emptying (**A**) versus normal gastric emptying (**B**) in solid gastric scintigraphy

The mean T_1_/_2_ of gastric emptying for all study population, GP group, and GP-like group were 105.21 ± 44.97, 129.78 ± 38.29, and 64.28 ± 15.54 min, respectively (p = < 0.001). Figure 3 illustrates delayed gastric emptying (T_1_/_2_: 193 min) compared to normal gastric emptying (T_1_/_2_: 60 min) in solid gastric scintigraphy studies in two different patients with severe symptoms.

The correlations between T_1_/_2_ of gastric emptying and other variables in the entire study population are shown in Table 3. Significant positive correlations were found between T_1_/_2_ of gastric emptying and serum creatinine, AST, the total GCSI, the average GCSI, nausea/vomiting subscale, and vomiting severity (*p* = 0.024, 0.006, 0.004, 0.009, 0.033, 0.030, respectively). A significant negative correlation was observed with hemoglobin levels (*p* = 0.004).

**Table 3:** The correlations between T_1_/_2_ of gastric emptying and other variables in all study population

Variables	T_1_/_2_ of gastric emptying		
	Correlation Coefficient	*P* value	*N*
Age (Y)	0.023	0.900	32
Duration of DM (Y)	0.216	0.235	32
BMI	-0.133-	0.467	32
HbA1c (%)	-0.007-	0.970	32
Hb	-0.511-	**0.004**	30
TLC	0.038	0.840	30
PLT	-0.063-	0.741	30
S. Creatinine	0.397	**0.024**	32
eGFR	-0.318-	0.076	32
AST	0.578	**0.006**	21
ALT	0.117	0.604	22
uACR	0.285	0.284	16
TC	-0.185-	0.329	30
LDL	-0.254-	0.255	22
HDL	-0.294-	0.268	16
TG	-0.052-	0.786	30
Symptoms Duration (M)	0.184	0.312	32
Total GCSI /45	0.491	**0.004**	32
Nausea/Vomiting subscale/5	0.378	**0.033**	32
Post prandial fullness/Early satiety subscale/5	0.197	0.281	32
Bloating/Distension subscale/5	0.319	0.075	32
Nausea	0.192	0.294	32
Retching	0.328	0.067	32
Vomiting	0.384	**0.030**	32
Stomach fullness	-0.009-	0.960	32
Not able to finish a meal	0.195	0.285	32
Excessive post-prandial fullness	0.179	0.328	32
Loss of appetite	0.056	0.760	32
Bloating	0.319	0.075	32
Visible abdominal distension	0.283	0.117	32

N Number, Y Years, DM Diabetes Mellitus, BMI Body Mass Index, Hb Hemoglobin, TLC Total Leukocyte Count, PLT Platelet Count, S. Creatinine Serum Creatinine, eGFR Estimated Glomerular Filtration Rate, AST Aspartate Aminotransferase, ALT Alanine Transaminase, uACR Urinary Albumin/Creatinine Ratio, TC Total Cholesterol, LDL Low-Density Lipoprotein, HDL High-Density Lipoprotein, TG Triglycerides, M Months, GCSI Gastroparesis Cardinal Symptom Index

### Response to medical treatment

**Fig. 4:**
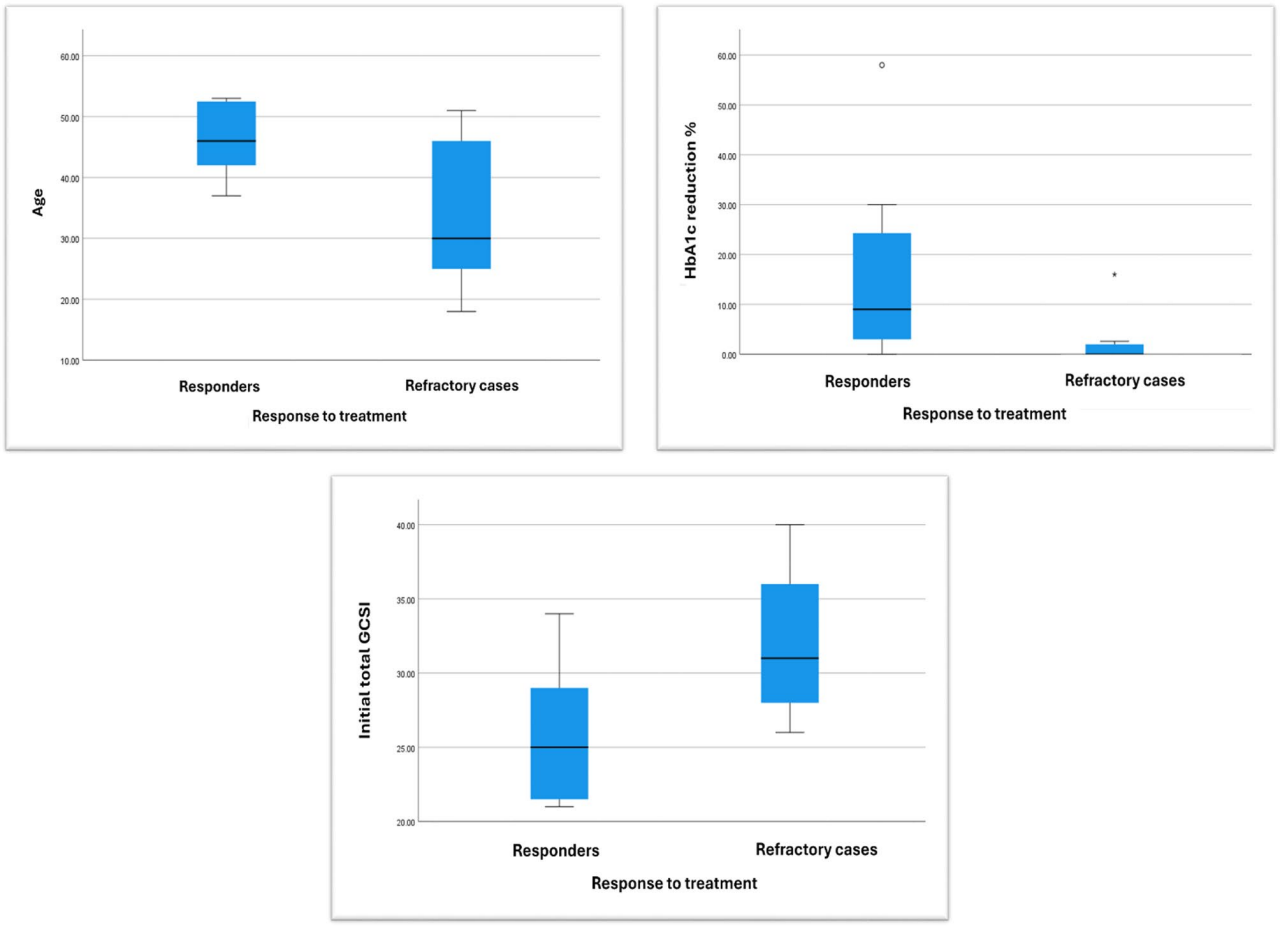
The correlations between Age, HbA1c reduction (%), initial total GCSI and response to medical treatment

**Fig. 5:**
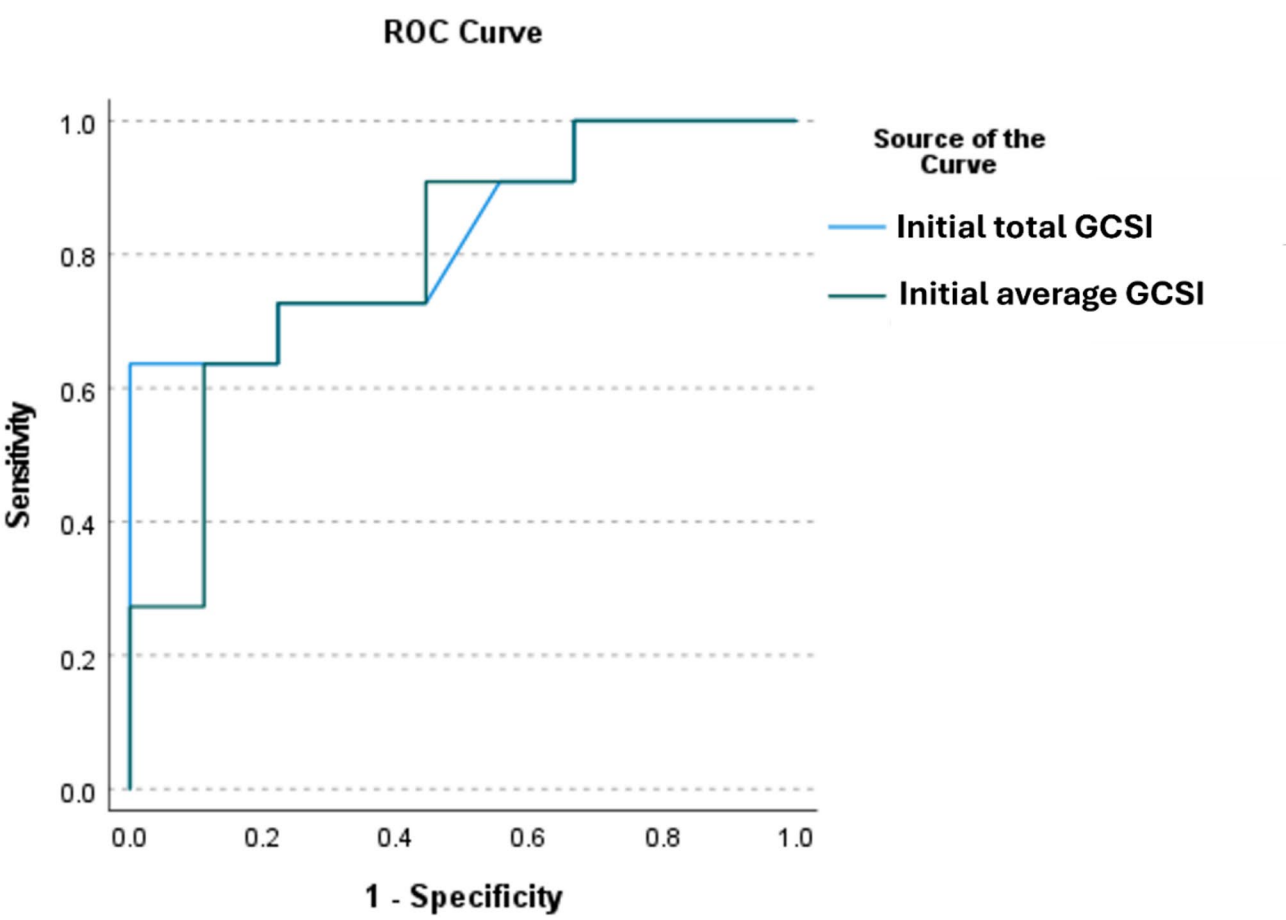
Sensitivity and specificity of the initial total and average GCSI in predicting response to medical treatment

After medical treatment of patients in the GP group for three months, 55% of cases reported improvement of symptoms. A comparison between the responders and refractory groups is shown in Table 4. Responders were significantly older than those in the refractory group (*p* = 0.046), and there was a significantly greater HbA1c reduction (%) in the responders’ group compared to the refractory group (*p* = 0.012). The mean HbA1c dropped from 9.88 to 8.08% in the responders and from 8.44% to 8.30 in the refractory group. Responders also had significantly lower total and average GCSI scores (*p* = 0.012, 0.025, respectively) compared to the refractory group (Fig. 4). Logistic regression analysis to identify independent predictors of response to medical treatment revealed that the initial total GCSI score was a predictor of the response to medical treatment (OR 0.764, 95% CI 0.57–0.977 and *p* = 0.032). An initial total GCSI cutoff value of < 25 was found to be 100% specific and 63.6% sensitive for predicting clinical response to medical treatment (AUC 0.828, *p* < 0.001, 95% CI 0.647–1.009) (Fig. 5).

**Table 4:** Comparison between responders’ and refractory groups

Variables	Responders		Refractory		*P* value
	Mean	SD	Mean	SD	
Age (Y)	46.09	6.11	34.11	12.62	**0.046**
Duration of DM (Y)	13.73	6.40	12.11	5.16	0.603
Baseline HbA1c	9.88	2.20	8.44	1.60	0.112
HbA1c after treatment	8.08	1.30	8.30	1.48	0.864
HbA1c reduction (%)	15.14	17.82	2.29	5.24	**0.012**
Duration of symptoms (M)	54.82	88.06	51.44	94.19	0.766
Initial total GCSI /45	25.64	4.59	32.00	5.27	**0.012**
Initial average GCSI /5	2.89	0.55	3.58	0.67	**0.025**
Nausea/Vomiting subscale/5	2.03	1.35	3.00	1.20	0.175
Post-prandial fullness/Early satiety subscale/5	3.14	0.96	3.75	0.68	0.152
Bloating/Distension subscale/5	3.50	1.43	4.00	1.44	0.295
Nausea	3.18	1.60	2.89	1.90	0.766
Retching	1.55	1.57	3.11	1.54	0.056
Vomiting	1.36	1.91	3.00	1.87	0.067
Stomach fullness	3.82	1.47	4.11	1.27	0.603
Not able to finish a meal	3.36	1.43	3.78	1.09	0.656
Excessive post-prandial fullness	3.09	1.81	3.56	1.33	0.656
Loss of appetite	2.27	1.90	3.56	1.51	0.152
Bloating	3.64	1.57	4.00	1.32	0.603
Visible abdominal distension	3.36	1.43	4.00	1.58	0.152

SD Standard Deviation, Y Years, GCSI Gastroparesis Cardinal Symptom Index, M Months, DM Diabetes Mellitus

### G-POEM group

Of the nine refractory cases, only four consented to undergo G-POEM. The mean age of participants was 38.50 ± 11.79 years, consisting of three females and one male, all had type 2 DM with a mean duration of 10.25 ± 3.69 years and fair glycaemic control. Two participants had diabetes-related complications, and the mean BMI was 28.73 ± 1.59 kg/m^2^. The mean initial total GCSI was 32.75 ± 7.8, with nausea and vomiting being the dominating symptoms. The mean T_1_/_2_ of gastric emptying was 166.25 ± 59.77 min. Two out of four patients achieved sustained clinical improvement one year after the procedure. In one of them T_1_/_2_ of gastric emptying showed 25% reduction after 3 months. Among the two patients who did not improve, one showed no change in gastric scintigraphy after 3 months. The mean reduction in the total GCSI in the four cases was 9.5 ± 5 points. Two out of the four patients achieved glycaemic control (HbA1c < 6.5%) after G-POEM and the mean reduction in HbA1c (%) was 1.98 ± 1.4. No complications were reported.

## Discussion

This study included thirty-two patients who presented with symptoms suggestive of gastroparesis. Among them, twenty patients (62.5%) had objectively confirmed delayed gastric emptying (GP group), while twelve patients (37.5%) had normal gastric emptying (GP-like group). The majority of patients were females (81.3%) with a mean age of 40.59 ± 11.13 years, aligning with the findings of Navas CM et al.; they reported a predominance of middle-aged females [7].

In this study, no significant differences were observed between the GP and GP-like groups regarding age, sex, type of DM, DM treatment and complications, HbA1c (%), smoking status, or comorbidities. Although not statistically significant, the GP group had a higher prevalence of diabetic nephropathy than the GP-like group (12 cases vs. 3 cases, *p* = 0.055). These findings are consistent with Bharucha et al., who reported delayed gastric emptying in 36% of their cohort (46 patients). They found no associations between gastric emptying and demographic features (age, sex, and BMI), smoking status, type and duration of DM, use of insulin, HbA1c (%), or the presence of diabetes-related complications [8].

Similarly, Navas CM et al. found no correlation between gastric emptying and the referring symptom, duration of DM, HbA1c (%), or diabetes complications, though they observed an association between more severe gastric emptying delay and insulin dependence (*p* = 0.046) [7].

Chedid V et al. found that 19.4% of symptomatic patients had delayed gastric emptying. They concluded that gastric emptying is not related to diabetes control nor the duration of diabetes [9]. In contrast, Izzy M et al. reported an increased incidence of gastroparesis in patients with worse HbA1c (%) [10]. Additionally, Bharucha et al. found that patients with delayed gastric emptying had a longer duration of DM, higher HgbA1c level, and higher prevalence of retinopathy [11]. This aligns with the findings of Hyett et al., who also found that patients with GP had a longer duration of DM when compared to patients with GP-like symptoms [12].

In this study, post-prandial fullness was the dominating symptom, while the most dominant GCSI subscale was bloating/distension. This contrasts with Navas CM et al., who reported nausea and upper abdominal pain as the most common symptoms, followed by vomiting and early satiety [7]. Similarly, in Chedid V et al. study, the most common presenting symptom was nausea and vomiting [9]. These discrepancies may be attributed to differences in population characteristics, underlying comorbidities, and potential regional variations in symptom perception and reporting.

In this study, significant positive correlations were observed between the T_1_/_2_ of gastric emptying and several parameters including serum creatinine, AST, total GCSI, average GCSI, nausea/vomiting subscale, and vomiting severity (*p* = 0.024, 0.006, 0.004, 0.009, 0.033, 0,030, respectively). A significant negative correlation was also found between the T_1_/_2_ of gastric emptying and hemoglobin levels (*p* = 0.004). These findings may suggest an association between delayed gastric emptying and the presence of fatty liver and diabetic nephropathy. The inverse relation between hemoglobin levels and the T_1_/_2_ of gastric emptying may reflect nutrient deficiencies in gastroparesis patients due to poor food intake. This is supported by Parkman HP et al.’s study which demonstrated that many patients with gastroparesis consume diets deficient in calories, carbohydrates, proteins, vitamins, and minerals [13].

In this study, symptoms of GP were significantly more severe in the GP group compared to the GP-like group according to the total GCSI (*p* = 0.021) and the average GCSI (*p* = 0.048). Logistic regression analysis identified the total GCSI score as an independent predictor of delayed gastric emptying in gastric scintigraphy (OR 1.153, 95% CI (1.009–1.317), *p* = 0.036). A total GCSI greater than 23 demonstrated a sensitivity of 80% and a specificity of 66.7% for identifying delayed gastric emptying (AUC 0.746, *p* = 0.016, 95% CI 0.545–0.947). These findings should be taken with caution, given the wide confidence interval and the small sample size. It may not apply universally before validation in larger cohorts.

Cassilly DW et al. found that nausea, inability to finish a normal-size adult meal, and post-prandial fullness sub-score were positively correlated to gastric retention at 2 h (*p* = 0.09 and *p* = 0.005, *p* = 0.01 respectively). The correlation between the total GCSI and gastric retention was significant at 2 h (correlation coefficient 0.144, *p* = 0.03) but not at 4 h (correlation coefficient 0.040, *p* = 0.55). Importantly, their logistic regression showed that none of the GCSI components independently predicted the diagnosis of gastroparesis, leading to the conclusion that the GCSI may not be a reliable predictor of gastroparesis among symptomatic patients [14]. Several other studies have similarly failed to identify a significant correlation between upper gastrointestinal symptom scores and gastric emptying [15–20]. Another recent study assessed whether the GCSI score could help in the diagnosis of gastroparesis, but did not find a clear diagnostic threshold [21]. Indeed, the main difference between the current study and the previous studies is that the current study only assessed diabetic patients, while previous studies mainly mixed diabetic and non-diabetic patients with gastroparesis. Interestingly, nausea and vomiting remained the symptoms with the strongest association with T_1_/_2_.

The management of gastroparesis is challenging and requires a multi-disciplinary approach. Potential mechanisms of response to medical treatment include: improved gastric motility due to better glycaemic control, neuro-modulatory effects of prokinetic agents, and discontinuation of DPP-4i/metformin combination that may contribute to gastrointestinal symptoms. In our study, 55% of cases responded to a three-month course of medical treatment. Navas CM et al. found that about 40% of cases reported improvement following anti-emetic therapy with domperidone and metoclopramide; however, they didn’t use GCSI to measure the severity of symptoms [7]. In a single-center cohort of 115 cases of GP (16 of whom had DM), domperidone therapy for an average of three months led to improvement in 69 patients (60%), and moderate improvement in 45 patients (39%), as assessed by the Clinical Patient Grading Assessment Scale [22]. In a study by Parkman HP et al.., including 48 GP patients, 81% of patients showed improvement after domperidone therapy [23]. Another study, which included 262 cases of GP diagnosed by solid GS (32% of whom had DM), assessed symptoms using the GCSI before and after 48 weeks of medical treatment. In this cohort, 15% of patients achieved a ≥ 50% improvement in their GCSI score [24].

In the current study, Responders were significantly older than those in the refractory group (*p* = 0.046). This is consistent with findings from Parkman et al.., who observed that patients < 45 years old had a significantly lower clinical response (1.18 ± 1.05; *n* = 22) compared to patients ≥ 45 years old (1.88 ± 0.80; ***n*** = 27; *p* < 0.05) [23]. Similarly, Pasricha PJ et al. reported that older age (≥ 50 years) was associated with the best outcome, with an odds ratio for improvement of 3.35 (CI:1.62–6.91, *p* = 0.001) [24]. These observations may be attributed in part to greater patient satisfaction in older individuals, and the subjective nature of symptom assessment scores.

In our study, Responders also had significantly lower initial total and average GCSI scores (*p* = 0.012, *p* = 0.025 respectively). This finding contrasts with the study by Pasricha et al.., where higher total GCSI scores were associated with a more favorable response to treatment after 48 weeks (OR = 2.87, CI: 1.57–5.23, p ***=*** 0.001) [24]. This discrepancy may reflect the unique profile of our cohort, in which severe symptoms are more likely to represent greater disease burden and therapeutic resistance. More severe symptoms may be indicative of greater autonomic dysfunction, and significant gastric dysmotility limiting the efficacy of standard medical treatment. This interpretation is further supported by findings from Amjad et al., where the presence of peripheral neuropathy was associated with treatment failure [25]. Additionally, the pathophysiological differences between diabetic and idiopathic gastroparesis may explain the divergence in findings between our study and Pasricha et al.’s, where two-thirds of patients were non-diabetic. It is possible that in idiopathic gastroparesis, symptom severity reflects a component of visceral hypersensitivity, which might respond differently to therapy compared to the predominant motility dysfunction seen in diabetic gastroparesis. These findings have important clinical implications. In diabetic gastroparesis, severe symptoms may indicate a higher likelihood of refractoriness to standard medical therapy. This suggests the need for stratified treatment approaches, where patients presenting with high symptom burdens may require alternative or more aggressive interventions, such as G-POEM to reduce unnecessary prolonged medical treatments in those unlikely to benefit.

In our study, a significantly greater HbA1c reduction (%) was reported in the responders’ group than those in the refractory group (*p* = 0.012). This underscores the potential role of glycaemic control in the management of gastroparesis and highlights the bi-directional relation between glycemia and gastroparesis. On one hand, gastroparesis worsens hyperglycemia due to poor oral intake and poor adherence to anti-diabetic medications, often due to post-prandial hypoglycemia. On the other hand, hyperglycemia itself has been shown to worsen gastroparesis [26]. While several studies have explored the impact of glycaemic control on GP severity, their findings have been inconsistent, highlighting the need for large-scale studies to investigate this relationship [27–30].

G-POEM is a promising new procedure for the management of refractory GP. According to the American Gastroenterology Association recommendations, G-POEM should be offered to adult patients with refractory gastroparesis who have gastric outlet obstruction been excluded by gastroduodenoscopy, have delayed gastric emptying in a solid gastric scintigraphy, and have moderate-to-severe symptoms preferably with nausea and vomiting as the dominant symptoms [6]. When performed by experienced endoscopists, G-POEM is generally safe, and complications are uncommon. However, serious complications have been reported like bleeding, perforation, capno-peritoneum, gastric ulceration, and dumping syndrome [31–33]. In the current study, only 4 of the 9 patients in the refractory group consented to undergo G-POEM. Sustained clinical improvement at 1 year was achieved in 2 cases only. While these results are preliminary and based on a small sample, they align with the existing literature. A systematic review assessing the 1-year clinical outcome after G-POEM reported a pooled clinical success rate of 61% (95% CI) and an adverse event rate of 8% [34].

### Limitations

This study has several limitations. First, the study population was drawn from a single diabetes clinic, which may limit the generalizability of the findings. Additionally, symptomatic patients who declined scintigraphy were not included, introducing potential selection bias. The relatively small sample size compared to other studies may also reduce the statistical power, particularly in the logistic regression analysis, making the results exploratory rather than conclusive. Another limitation is that upper endoscopy was not performed in all patients to exclude potential gastric outlet obstruction. Furthermore, blood glucose levels were not measured immediately prior to gastric scintigraphy. Given that hyperglycemia can delay gastric emptying, this could have influenced the gastric emptying parameters. Additionally, the use of gastric emptying T_1_/_2_ as the primary scintigraphic parameter, rather than the standard 4-hour retention values, is another limitation. The follow-up period was limited to three months, which was sufficient for short-term assessment of symptom response but may not fully capture the fluctuating nature of gastroparesis. Future studies are needed to assess the long-term outcomes and to determine whether patients in the GP-like group eventually develop gastroparesis. Another limitation of this study is the lack of a validated Arabic version of the GCSI questionnaire. While we verbally translated the questionnaire to facilitate patient understanding, the absence of a standardized linguistic and cultural validation process may have affected the consistency of symptom scoring. Future studies should consider using a formally validated Arabic translation to improve the accuracy of patient-reported outcomes.

## Conclusion

The current study highlighted the role of the GCSI in assessing and monitoring gastroparesis, particularly in resource-limited settings. Diabetic GP differs in clinical presentation and management from other types of GP. Optimizing glycemic control may contribute to symptoms improvement. Older age and lower symptoms burden at presentation may help identify patients more likely to benefit from medical therapy. As this is an exploratory study, larger and well-designed studies are needed to confirm our findings.

### Recommendations

Further larger studies are needed to confirm our findings.

## Data Availability

All data generated or analyzed during the current study are included in the published article.

## Abbreviations

BMI: Body Mass Index
DPP-4i: Di-Peptidyl: Peptidase 4 Inhibitors
DM: Diabetes Mellitus
eGFR: Estimated Glomerular Filtration Rate
GCSI: Gastroparesis Cardinal Symptom Index
GP: Gastroparesis
G-POEM: Gastric Per-Oral Endoscopic Myotomy
GS: Gastric Scintigraphy
Half-time: T_1_/_2_
HbA1c: Hemoglobin A1c
PPIs: Proton Pump Inhibitors
